# Isolation of a 3-hydroxypyridine degrading bacterium, *Agrobacterium* sp. DW-1, and its proposed degradation pathway

**DOI:** 10.1186/s13568-019-0782-9

**Published:** 2019-05-17

**Authors:** Shuxue Zhao, Chunhui Hu, Lizhong Guo, Kuiran Li, Hao Yu

**Affiliations:** 1 Shandong Provincial Key Laboratory of Applied Mycology, College of Life Sciences, Qingdao Agricultural University, 700 Changcheng Road, Chengyang District, Qingdao, 266109 Shandong Province People’s Republic of China; 2 Key Laboratory of Marine Environmental Science and Ecology, Ministry of Education, Ocean University of China, 238 Songling Road, Laoshan District, Qingdao, 266100 Shandong Province People’s Republic of China

**Keywords:** 3-Hydroxypyridine, Microbial degradation, *Agrobacterium*, Pathway, 2,5-Dihydroxypyridine

## Abstract

A 3-hydroxypyridine degrading bacterium, designated strain DW-1, was isolated from petroleum contaminated soil in Liao River China. 16S rRNA-based phylogenetic analysis indicates that strain DW-1 belongs to genus *Agrobacterium*. The optimal cultivation temperature and pH for strain DW-1 with 3-hydroxypyridine were 30 °C and 8.0, respectively. Under optimal conditions, strain DW-1 could completely degrade up to 1500 mg/L of 3-hydroxypyridine in 66 h. The 3-hydroxypyridine degradation pathway of strain DW-1 was suggested by HPLC and LC–MS analysis. The first reaction of 3-hydroxypyridine degradation in strain DW-1 was *α*-hydroxylation so that the major metabolite 2,5-dihydroxypyridine was produced, and then 2,5-dihydroxypyridine was transformed by a Fe^2+^-dependent dioxygenase to form *N*-formylmaleamic acid. *N*-Formylmaleamic acid will be transformed to maleic acid and fumaric acid through maleamic acid. This is the first report of the 3-hydroxypyridine degradation pathway and the utilization of 3-hydroxypyridine by a *Agrobacterium* sp. It may be potentially used for the bioremediation of environments polluted with 3-hydroxypyridine.

## Introduction

Pyridine and its derivatives compose one of the largest classes of *N*-heterocyclics (Fetzner 1998; Kaiser et al. 1996; O’Hagan 2000; Scriven and Murugan 1996; Sims et al. 1989). They were mainly produced by mining industry, petroleum industry and chemical synthesis industry. The nitrogen atom makes pyridine compounds variety structures and outstanding biological activities (Ahmed et al. 2019; Fetzner 1998; Han et al. 2017). Therefore, pyridine derivatives are one of the most important privileged compounds in organic chemistry, that find application in medicinal drugs and in agricultural products, such as herbicides, insecticides, fungicides, and plant growth regulators (Ahmed et al. 2019; Han et al. 2017). Because of their heterocyclic structures, pyridinics are more soluble in water than their homocyclic analogs and can be easily transported to groundwater, which may cause serious implications for human health. Besides, the aromatic-like structure makes these compounds recalcitrant to degradation. Therefore, pyridine and its derivatives are classified as priority pollutants by the United States Environmental Protection Agency due to their carcinogenicity and toxicity (Kuhn and Suflita 1989; Richards and Shieh 1986; Sims et al. 1989). While pyridinic compounds can be removed by physical and chemical methods, bioremediation is a less costly alternative approach to clean up the environment polluted with pyridine compounds without secondary pollution (Shi et al. 2018; Zheng et al. 2017).

3-Hydroxypyridine is an important pyridine derivative, which is widely used as precursor for the synthesis of medicines, daily chemicals, and pesticides (Garcia Linares et al. 2012; Sabot et al. 2012), such as trifloxysulfuron and pirbuterol. Its soluble properties make 3-hydroxypyridine easy to spread in the environment, which may do great damage to the environment. Till now, only a few strains/bacterial consortium capable of degrading 3-hydroxypyridine have been isolated and characterized (Cain et al. 1974; Houghton and Cain 1972; Kaiser and Bollag 1991). The reported strains were belong to the genera of *Achromobacter* and *Nocardia*. The knowledge gap exists on biodegradation of 3-hydroxypyridine with new strains currently. Although a few intermediates have been reported during 3-hydroxypyridine degradation, the complete degradation pathway of 3-hydroxypyridine remains enigmatic (Cain et al. 1974; Kaiser et al. 1996). Deciphering the degradation pathway of 3-hydroxypyridine is of scientific significant since it will be helpful for the microbial degradation of 3-hydroxypyridine and related pollutants.

So far, researches have not reported the capacity of bacteria on removal of 3-hydroxypyridine from water, and the microbial degradation pathway of 3-hydroxypyridine was remain unclear. To fill the knowledge gap, a new 3-hydroxypyridine strain, *Agrobacterium* sp. DW-1, was isolated and the aerobic degradation of 3-hydroxypyridine were described. The intermediates and enzyme activity were detected to dissect the degradation pathway of 3-hydroxypyridine in strain DW-1. To our knowledge, this is the first report on the degradation of 3-hydroxypyridine by a *Agrobacterium*.

## Materials and methods

### Chemicals

3-Hydroxypyridine, 2-hydroxypyridine, 4-hydroxypyridine, methylpyridine was purchased from Aladdin Co. (Shanghai, China) and were analytical grade. 3,4-Dihydroxypyridine, 3,5-dihydroxypyridine and 3-pyridinol *N*-oxide were purchased from Sigma-Aldrich (United States). All organic solvents were chromatographically grade. All other chemicals used in this study were commercial available and of analytical grade. The mineral salt medium (MSM), as described previously by Yu et al. (2018), was used in the cultivation and biodegradation experiments. The strain DW-1 was grown in 250-mL Erlenmeyer flasks containing 50 mL MSM medium with 1000 mg/L 3-hydroxypyridine incubated at 30 °C on shaker (150 rpm). 3-Hydroxypyridine was added before autoclaving. Other pyridine compounds were added before inoculation and filtered by a 0.22 μm filter.

### Isolation and identification of strain DW-1

The bacterial strain used for degradation was isolated from the petroleum contaminated soil from wetland of Liao River (Liaoning Province, China). Five gram of soil was added to 50 mL sterilized MSM medium with 1000 mg/L 3-hydroxypyridine, 1000 mg/L yeast extract and 1000 mg/L beef extract in a 250 mL flask for enrichment culture at 30 °C under aerobic condition in a rotary shaker. When the culture became obviously turbid, 5 mL of the culture was transferred into 50 mL sterilized MSM medium with 1000 mg/L 3-hydroxypyridine and cultured under the same condition for selective cultivation. The 16S rRNA gene of strain was amplified with universal primers 27F and 1492R with the genome as the template. The PCR reaction was performed using the following cycling conditions: 95 °C for 5 min, 35 cycles of 95 °C for 30 s, 58 °C for 30 s and 72 °C for 90 s, followed by 72 °C for 10 min. The PCR product was sequenced in Sangon Biotech (Shanghai) Co., Ltd. Sequence alignment was performed by using ClustalX software, and the phylogenetic tree was constructed by Mega 6.0 software. Scanning electron microscope (SEM) analysis was performed on JSM-7500F (JEOL, Japan).

### Growth and 3-hydroxypyridine degradation

The effect of pH on cell growth of strain DW-1 and degradation of 3-hydroxypyridine was assessed by cultivating the strain at various pH (pH 7.0–10.0) at 30 °C. The effect of temperature on cell growth of strain DW-1 was determined by cultivating the strain at different temperatures in the range from 25 to 37 °C. Then, the growth and degradation of strain DW-1 under optimal conditions were detected with different concentration of 3-hydroxypyridine. The cell density was determined according to the absorbance at 600 nm, and 3-hydroxypyridine concentration was monitored using high performance liquid chromatography (HPLC).

### Resting cells reactions

To prepare resting cells, strain DW-1 was cultivated in MSM media with 1000 mg/L 3-hydroxypyridine or 1000 mg/L glycerol + NH_4_Cl. The cells of strain DW-1 were collected in the late exponential phase by centrifugation at 8000×*g* for 5 min. The cells were washed twice with potassium phosphate buffer (50 mM, pH 7.0), and the precipitated cells were re-suspended with the same buffer and designated as resting cells. Resting cells reactions were performed in a 150 mL Erlenmeyer flask with 30 mL resting cells and 1000 mg/L 3-hydroxypyridine at 30 °C 150 rpm. Samples of the resting cells reactions were withdrawn at regular time intervals by centrifugation at 10,000×*g* for 2 min, and the supernatant was transferred to a new tube and stored at − 20 °C for further analysis.

To detect the enzyme activity, cells of strain DW-1 cultivated with 3-hydroxypyridine were collected by centrifugation. Collected cells were broken by ultrasonication and centrifuged at 10,000×*g* for 10 min. The supernatant was transferred to new tube as cell extract for enzyme activity detection. The 2,5-dihydroxypyridine dioxygenase activity was monitored according to the absorbance decrease at 320 nm in 50 mM Tris–HCl buffer (pH 8.0) at room temperature (25 °C) using Biophotometer Plus (Eppendorf). The reactions were performed in a 1 mL cuvette with a reaction volume of 800 μL. 250 μM 2,5-dihydroxypyridine (with/without 5 μM FeSO_4_) was added into the cuvette, and the enzyme assays were initiated by the addition of cell extract.

### Analytical methods

The ultraviolet spectrum scanning was performed on the Agilent Cary60 spectrophotometer. 3-Hydroxypyridine and the degradation intermediates were determined by HPLC with diode array detection, using reverse-phase column (Welch Xtimate C18, 4.6 mm × 250 mm, 5 μm) at 30 °C. The mobile phase was 20% (v/v) methanol and 80% distilled water at a flow rate of 1.0 mL/min. Mass spectrometry (MS) analysis was performed on an Orbitrap Fusion Lumos Tribrid (Thermo Fisher) equipped with electrospray ionization (ESI) sources, using reverse-phase column (Agilent ZORBAX RRHD Eclipse Plus 95Å C18 2.1 × 100 mm, 1.8 µm). The mobile phase contained 80% (v/v) 0.05% (w/v) formic acid and 20% (v/v) methanol at a flow rate of 0.2 mL/min. Both positive and negative electrospray ionization analysis with the continuous full scanning from *m/z* 50 to 500 were collected. Samples for HPLC and LC–MS analysis were treated by adding 9 volume of methanol and centrifuged at 10,000×*g* for 5 min, and then the supernatant was filtered by a 0.22 μm filter.

Nucleotide sequence accession numbers: The 16S ribosomal RNA gene sequence of *Agrobacterium* sp. DW-1 is available in GenBank under Accession Number MK402166.

## Results

### Isolation and characterization of strain DW-1

**Fig. 1:**
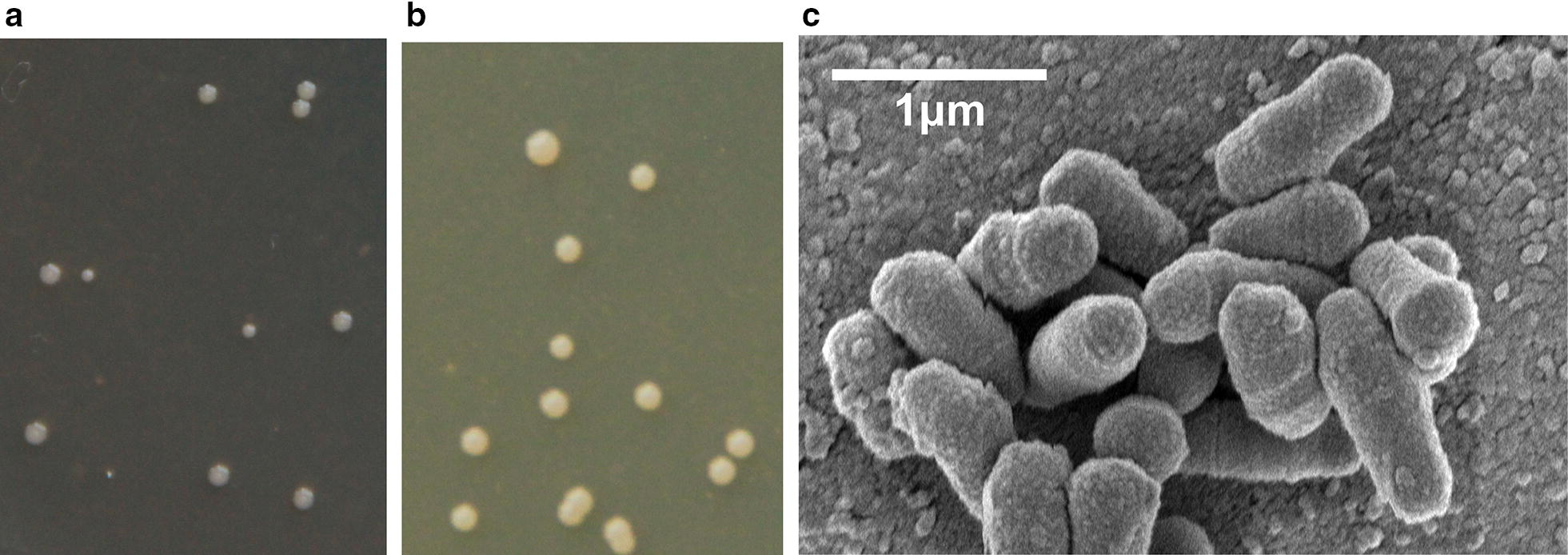
Colonies and scanning electron micrograph of strain DW-1. **a** Colonies of strain DW-1 grown in MSM agar plate with 3-hydroxypyridine. **b** Colonies of strain DW-1 grown in LB agar plate. **c** Scanning electron micrograph of cells of strain DW-1. Bar, 1 μm

**Fig. 2:**
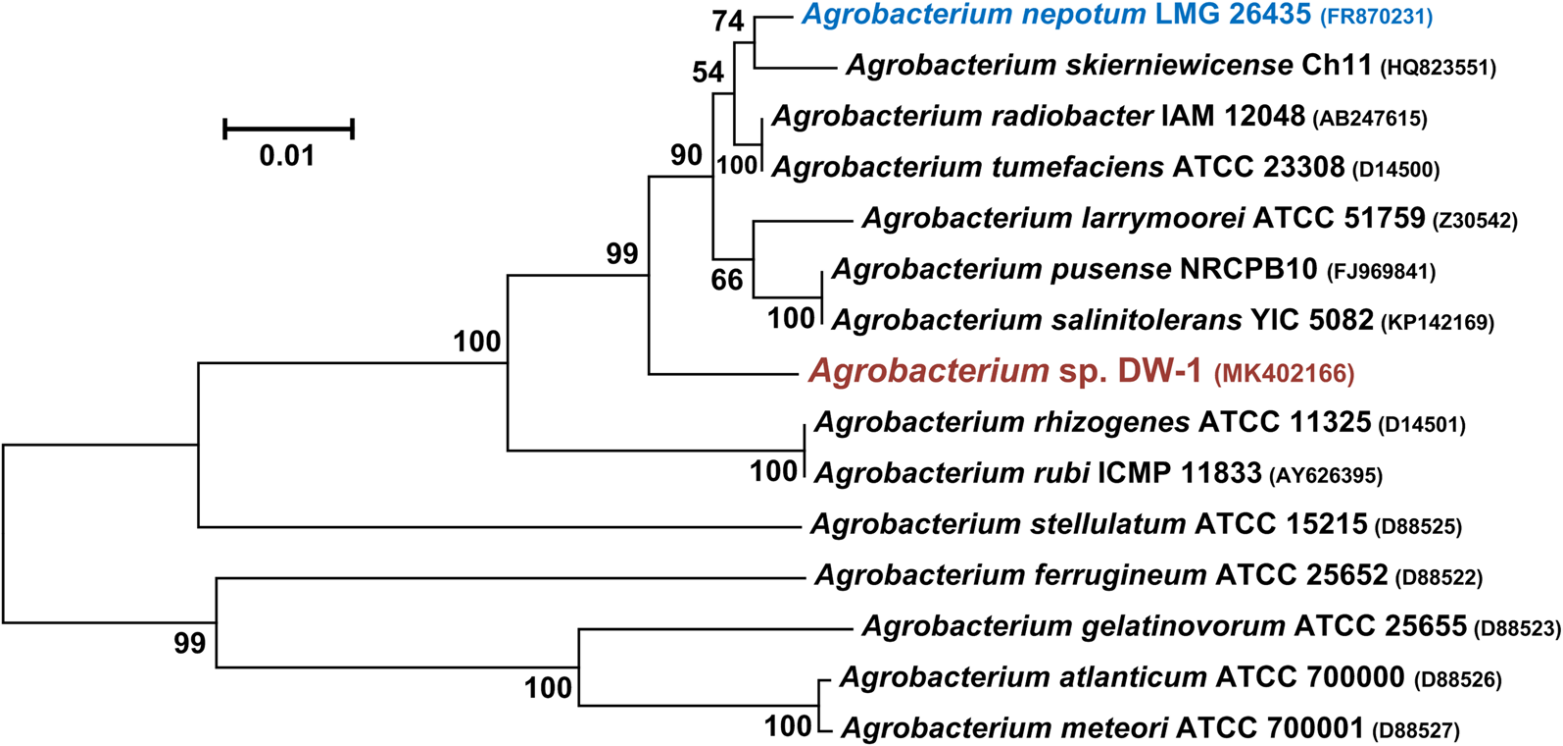
Neighbour-joining tree expression the relationships among *Agrobacterium* sp. DW-1 and the relatives, based on 16S rRNA sequences. Horizontal branch lengths are proportional to the estimated number of nucleotide substitutions and bootstrap probabilities (as percentages) are determined from 1000 resamplings

Strains were isolated from petroleum contaminated soil from Liao River. A dominant colony was selected from MSM agar plate with 3-hydroxypyridine as growth substrate by series dilution. The strain could utilize 3-hydroxypyridine as the sole source of carbon, nitrogen and energy, and was named DW-1. The colony of strain DW-1 was circular, convex, translucent to opaque, white or cream color, with a diameter of 0.5–1.5 mm within 2–4 days on MSM plate with 3-hydroxypyridine at 30 °C (Fig. 1a). The colony of strain DW-1 in LB plate was cream color with a diameter of 1–3 mm within 2 days (Fig. 1b). Cells of strain DW-1 were Gram-stain-negative, aerobic and non-spore forming rods (0.4–0.6 μm × 0.8–1.2 μm) (Fig. 1c). A comparison of the 16S rRNA gene sequence of strain DW-1 with those of other members of the genus *Agrobacterium* was performed based on the neighbour-joining methods (Fig. 2). Strain DW-1 exhibited the highest similarity (96.9%) to *Agrobacterium nepotum* LMG 26435, which is higher than any other genus. Therefore, strain DW-1 was identified as *Agrobacterium* sp. Several pyridinic compounds were tested as growth substrates for strain DW-1. DW-1 could only use 3-hydroxypyridine, and it could not use pyridine, 2-hydroxypyridine, 4-hydroxypyridine, 2,3-dihydroxypyridine, 3,4-dihydroxypyridine and methylpyridine as the growth substrate. *Agrobacterium* sp. DW-1 can be obtained from China general microbiological culture collection center under Accession Number of CCTCC M 2018821.

### Cell growth of strain DW-1 with 3-hydroxypyridine under different conditions

**Fig. 3:**
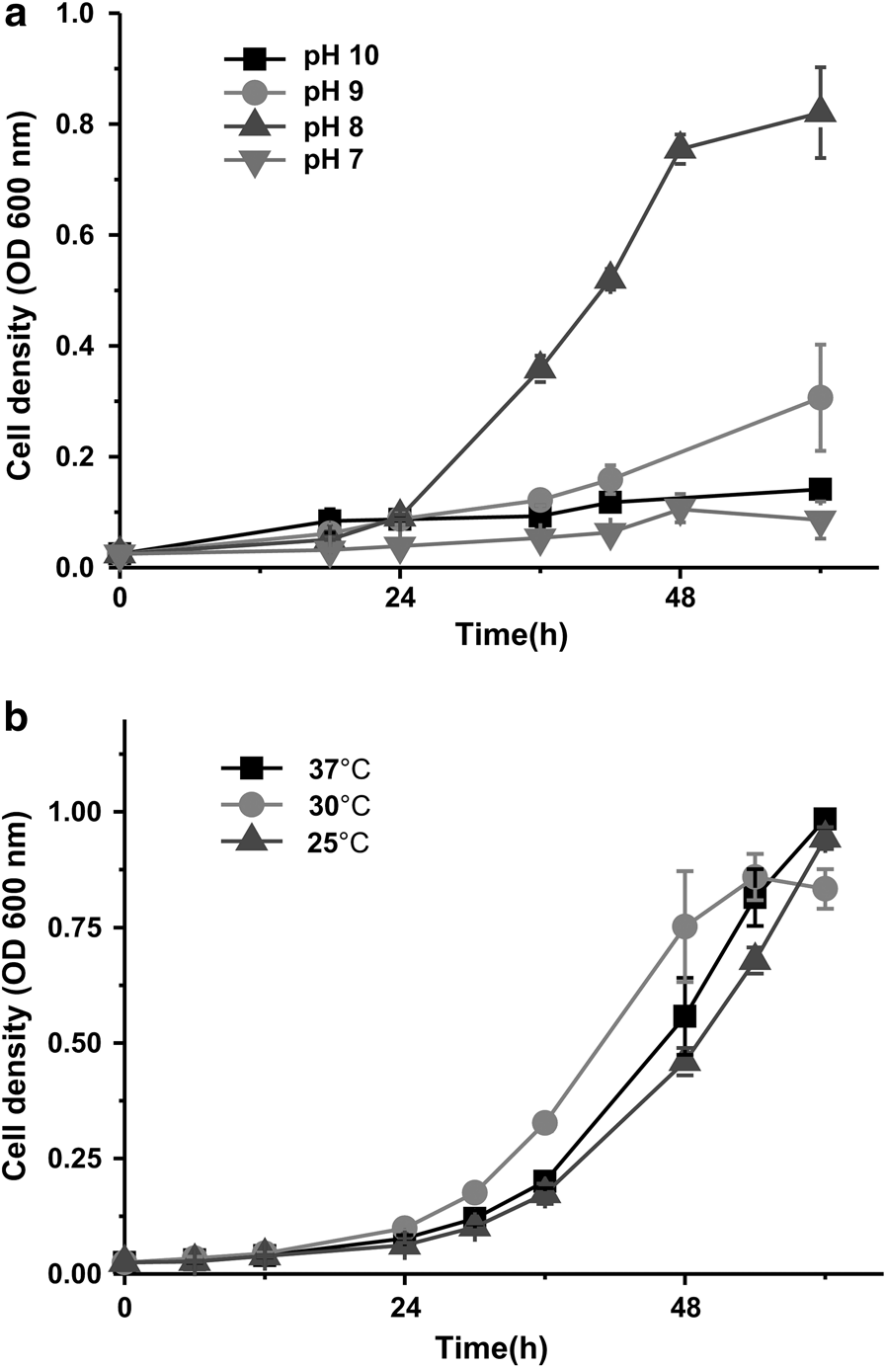
Effect of pH and temperature on cell growth of strain DW-1 in MSM with 3-hydroxypyridine. **a** pH dependent growth of strain DW-1. **b** Temperature dependent growth of strain DW-1. Each value is the mean from three parallel replicates ± SD

To obtain the optimum conditions under which strain DW-1 can most efficiently breakdown 3-hydroxypyridine, strain DW-1 was cultivated in MSM containing 3-hydroxypyridine with different pH and temperatures. As presented in Fig. 3a, it can be concluded that pH was a sensitive factor for cell growth of strain DW-1. The optimum pH value was pH 8.0; however, the lag phase was significantly extended at pH 7.0, 9.0 or 10.0 (Fig. 3a). The possible reason is that enzymes involved in 3-hydroxypyridine metabolism are sensitive to pH. By contrast, temperature has slight effect on cell growth of strain DW-1, and strain DW-1 grew well with all the tested temperature ranged from 25 to 37 °C (Fig. 3b). The optimal temperature is 30 °C due to that strain DW-1 has the shortest lag phase at this temperature.

### 3-Hydroxypyridine degradation

**Fig. 4:**
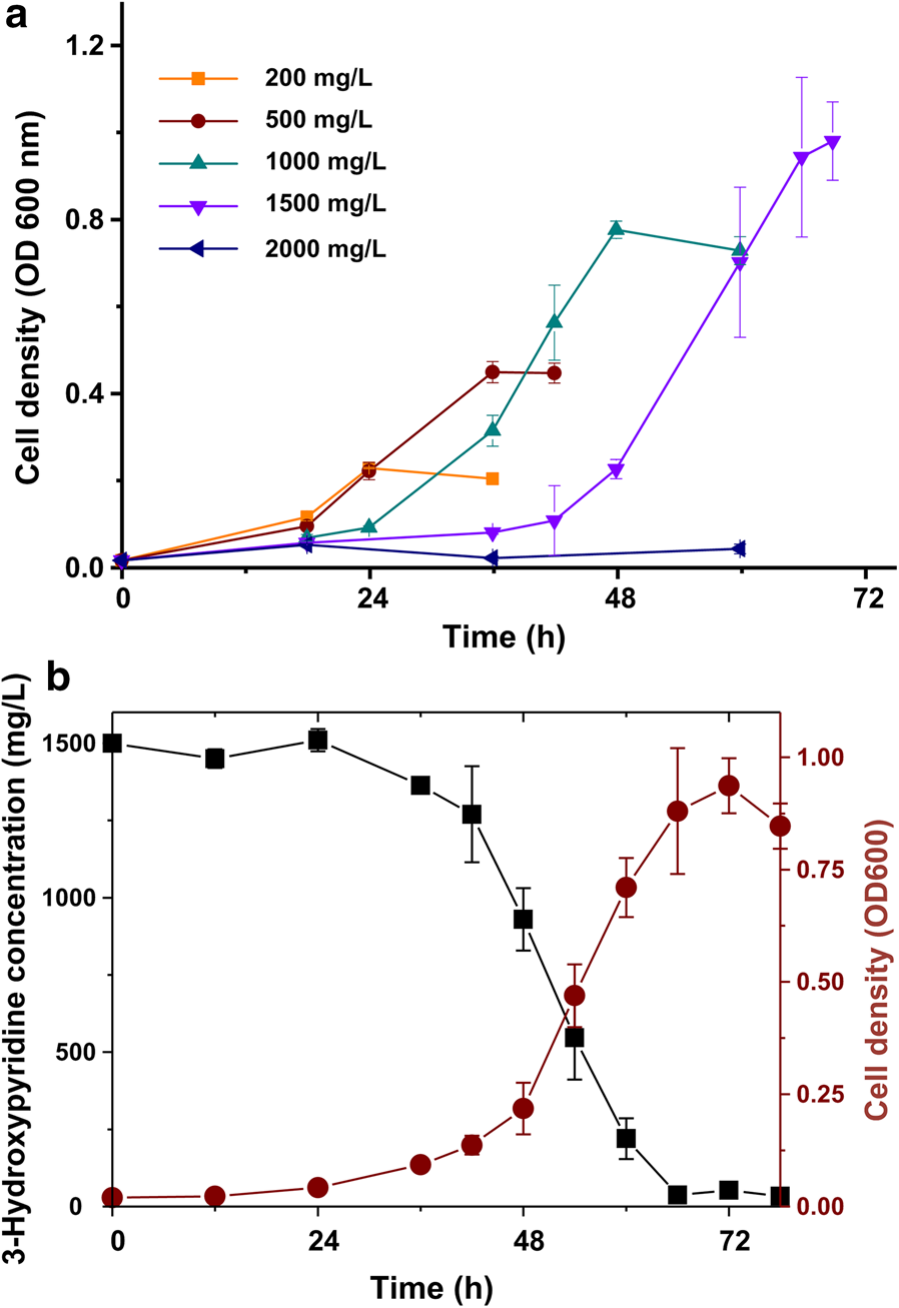
Effect of 3-hydroxypyridine concentration on cell growth and degradation of strain DW-1. **a** Growth of strain DW-1 with different concentration of 3-hydroxypyridine. **b** 3-Hydroxypyridine degradation by strain DW-1 with 1500 mg/L initial concentration. Each value is the mean from three parallel replicates ± SD

For the biodegradation experiment with different initial 3-hydroxypyridine concentration (in the range of 200–2000 mg/L), Fig. 4a shows the growth of strain DW-1. Strain DW-1 could grow on 3-hydroxypyridine at a initial concentration up to 1500 mg/L. The maximum cell density increased with the increase of 3-hydroxypyridine concentration from 200 to 1500 mg/L. However, when the initial concentration of 3-hydroxypyridine reached 2000 mg/L, strain DW-1 grew very slowly. Besides, the lag phase extended with the increase of 3-hydroxypyridine concentration from 500 to 1500 mg/L, indicating the inhibitory effect of 3-hydroxypyridine for the growth of strain DW-1. Figure 4b shows the changes in microbial cell density and residual 3-hydroxypyridine concentrations in batch culture of strain DW-1 under optimal condition. After a 24-h lag period, the cell density of strain DW-1 increased gradually from 0.04 to 0.88, and the 3-hydroxypyridine concentration decreased from 1500 to 30 mg/L within 42 h of incubation. The 3-hydroxypyridine removal rate was 98%, which indicated that 3-hydroxypyridine was completely mineralized.

### Degradation pathway of 3-hydroxypyridine in strain DW-1

**Fig. 5:**
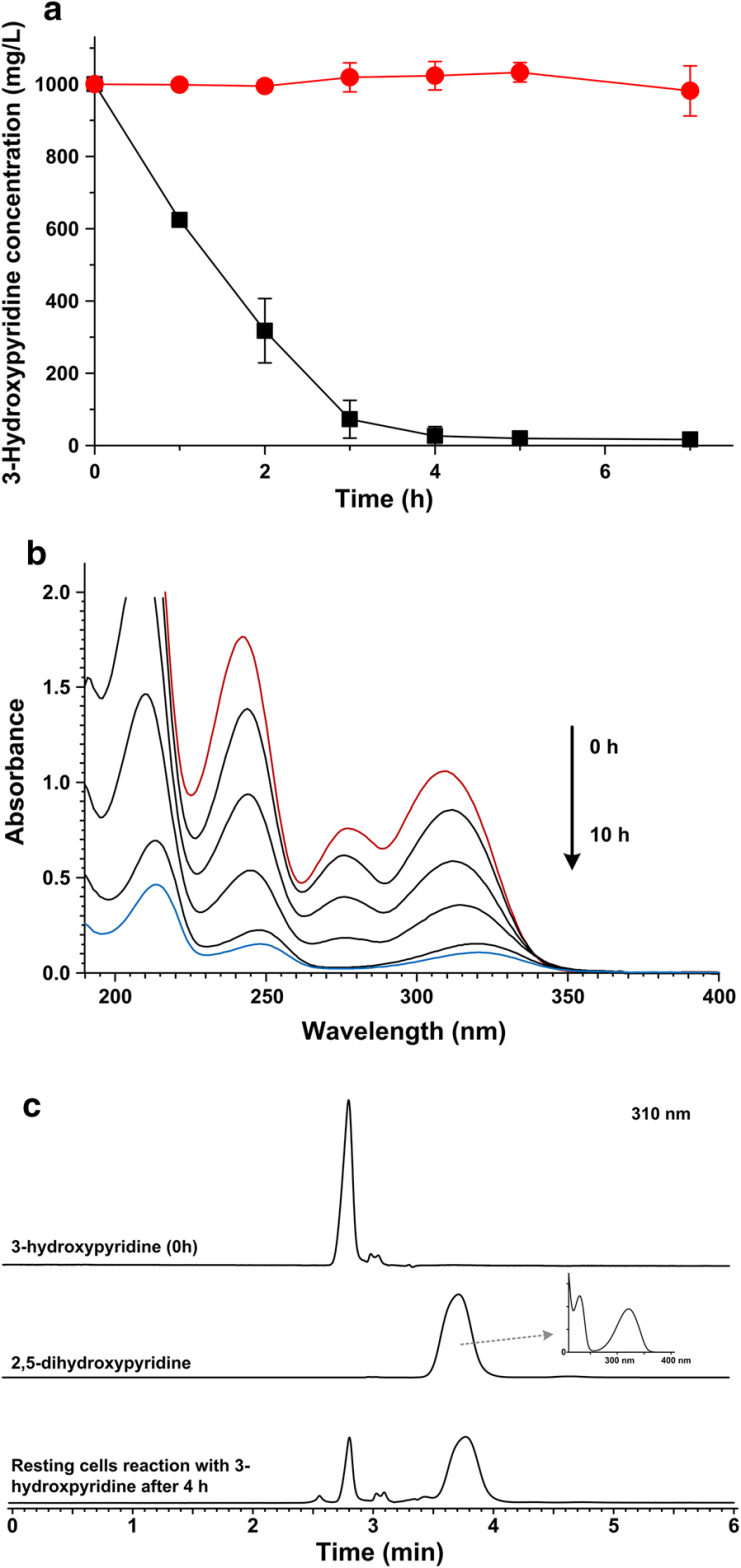
Analysis of 3-hydroxypyridine degradation by resting cells of strain DW-1. **a** 3-Hydroxypyridine degradation by resting cells of strain DW-1 grown in MSM with 3-hydroxypyridine (black line) or MSM with glycerol + NH_4_Cl (red line). **b** Ultraviolet spectrum scanning of samples from resting cells reactions. **c** HPLC analysis of samples from resting cells reactions

The degradation of pyridine derivatives by strain DW-1 was studied by using resting cells reactions. Resting cells of strain DW-1 could not transform 2/4-hydroxypyridine, 3,4-dihydroxypyridine, or 2,3-dihydroxypyridine. Cells cultivated with 3-hydroxypyridine or glycerol/NH_4_Cl were used to degrade 3-hydroxypyridine, respectively. It turned out that resting cells of strain DW-1 cultivated with 3-hydroxypyridine have far higher degradation rate than resting cells that cultivated without 3-hydroxypyridine (Fig. 5a). The results indicated that the expression of enzyme involved in 3-hydroxypyridine transformation was inducible, and 3-hydroxypyridine was the inducer. The ultraviolet spectrum scanning revealed that the absorbance of 3-hydroxypyridine decreased with the reaction time. The peak at 278 nm disappeared and the peak at 310 nm shifted to 320 nm, which is the characteristic peak of 2,5-dihydroxypyridine (Fig. 5b). 3-Hydroxypyridine transformation by strain DW-1 was analyzed by HPLC. When the signal representing 3-hydroxypyridine decreased, a new peak (with a retention time of 3.68 min) emerged in the HPLC signal. The retention time and spectra of the new product resemble those of 2,5-dihydroxypyridine (Fig. 5c) instead of 2,3-dihydroxypyridine, 3,4-dihydroxypyridine, 3,5-dihydroxypyridine, or 3-hydroxypyridine-1-oxide. No other peak was observed in the HPLC signal, which indicated that the 2,5-dihydroxypyridine was the ring-cleavage substrate, or the intermediates were transformed too fast to be detected.

**Fig. 6:**
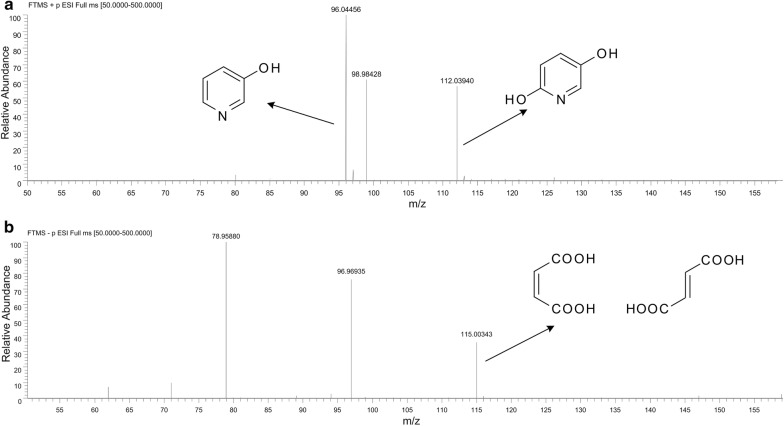
LC–MS analysis of 3-hydroxypyridine degradation intermediates by strain DW-1. **a** LC-MS signal of 3-hydroxypyridine and 2,5-dihydroxypyridine. **b** LC-MS signal of maleic acid and fumaric acid

To further investigate the degradation pathway of strain DW-1, the samples from resting cells reactions were detected using LC–MS. A spectrum with *m/z* of 96.04456 was observed, which represents a molecular formula of C_5_H_5_NO, and was identified as 3-hydroxypyridine. The peak with *m/z* of 112.03940 in the positive ion mode was identified as 2,5-dihydroxypyridine, which matched with formula C_5_H_5_NO_2_ (Fig. 6a). The signal of 2,5-dihydroxypyridine in resting cells reaction with 3-hydroxypyridine induced cells was ~ 70 fold high than that with 3-hydroxypyridine uninduced cells. A peak with a *m/z* of 115.00343 with a retention time of 1.255 min was observed (Fig. 6b). This mass corresponds with the molecular formula of C_4_H_4_O_4_, which was identified as maleic acid or fumaric acid.

### 2,5-Hydroxypyridine was transformed to *N*-formylmaleamic acid

**Fig. 7:**
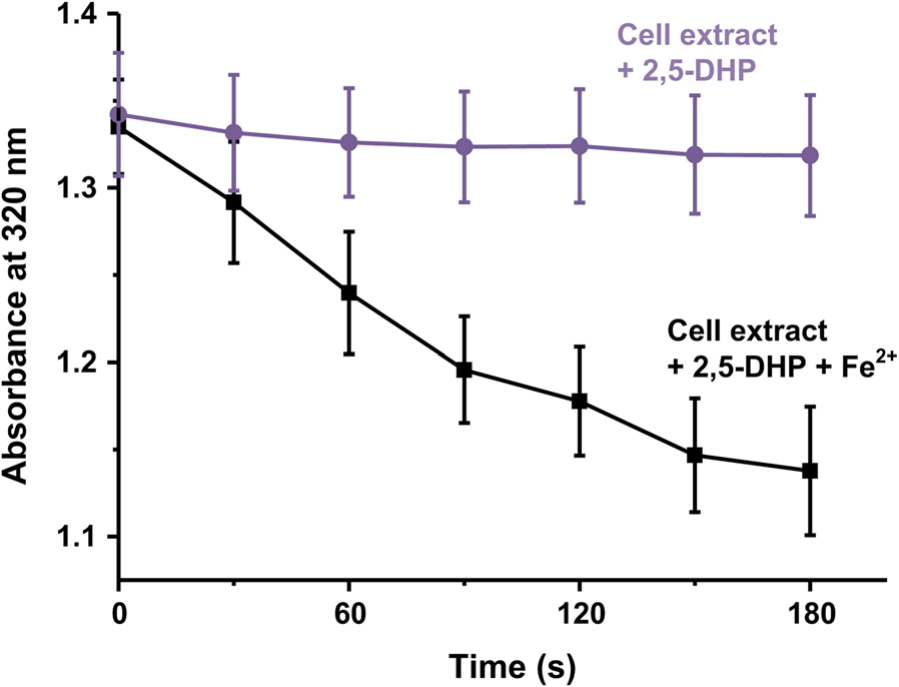
2,5-Dihydroxypyridine transformation by cell extract of strain DW-1. The reaction volume was 800 μL, in which 300 μL cell extract of strain DW-1 was mixed with 250 μM 2,5-dihydroxypyridine with or without 5 μM FeSO_4_

2,5-Dihydroxypyridine is the key metabolic intermediate of many pyridine derivatives, which is due to that 2,5-dihydroxypyridine is one of the important ring-cleavage intermediates. To identified the role of 2,5-dihydroxypyridine in strain DW-1, the 2,5-dihydroxypyridine dioxygenase activity was detected. The absorbance at 320 nm decreased with the addition of cell extract and 2,5-dihydroxypyridine, and the color of the reaction mixture was not changed. Moreover, the absorbance at 320 nm decreased rapidly with the addition of ferrous iron (dissolved in vitamin C solution) (Fig. 7). The results indicated that the 2,5-dihydroxypyridine was break by an iron^(II)^-dependent dioxygenase. The result was further confirmed by HPLC. In the HPLC signal, the peak representing 2,5-dihydroxypyridine disappeared after the reaction.

## Discussion

In the present study, a novel *Agrobacterium* sp. strain DW-1 capable of efficiency degradation of 3-hydroxypyridine was isolated from petroleum contaminated soil. So far, little has been reported about the capacity of microbial removal of 3-hydroxypyridine. Although several *Agrobacterium* strains capable of degrading pyridine derivatives have been studied, 3-hydroxypyridine degradation by a *Agrobacterium* strain has not been reported (Wang et al. 2012; Watson et al. 1974). Only a bacterial consortium in sewage sludge has been reported. The metabolism of 42.8 mg/L 3-hydroxypyridine in the sewage sludge required a lag period of 3–4 days, and the 3-hydroxypyridine was completed degraded for 6 days (Kaiser and Bollag 1991). By contrast, *Agrobacterium* sp. strain DW-1 showed better 3-hydroxypyridine degradation capacity. It could completely remove 1500 mg/L 3-hydroxypyridine within 66 h, with a lag period of 24 h. In general, the strain with high degrading capacity can enhance the degradation capacity of the bioreactor through bioaugmentation (Hou et al. 2018; Wen et al. 2013; Zhang et al. 2018). Therefore, strain DW-1 is a promising tool for treatment of 3-hydroxypyridine pollution.

Elucidation of the degradation pathway of pollutant is important for safe disposal. Microbial degradation of pyridine and its derivatives have been studied for decades, even though we have only deciphered the microbial degradation of a few compounds in the molecular mechanism level, including 2-hydroxypyridine, nicotinic acid, nicotinamide, 2-picolinic acid and nicotine (Hu et al. 2019; Jimenez et al. 2008; Qiu et al. 2019; Tang et al. 2013; Vaitekūnas et al. 2015; Yu et al. 2015). All the reported pyridine derivatives were degraded through two central intermediate 2,5-dihydroxypyridine, such as nicotinic acid, nicotinamide, 2-picolinic acid and nicotine (Hu et al. 2019; Jimenez et al. 2008; Qiu et al. 2019; Tang et al. 2013), and 2,3,6-trihydroxypyridine, such as 2-hydroxypyridine and nicotine (Vaitekūnas et al. 2015; Yu et al. 2015). At least two hydroxyl groups are needed for the ring cleavage reaction; therefore, hydroxylation of pyridine ring is the most common mode of initial attach of pyridine derivatives during microbial degradation. Three pyridine ring hydroxylation reactions have been reported including pyridine *α*-hydroxylation, pyridine *β*-hydroxylation and pyridine di-hydroxylation reactions. No pyridine *γ*-hydroxylation has been reported. Pyridine *α*-hydroxylation reaction, adding a hydroxyl group to the *α*-position of pyridine ring, is usually catalyzed by a molybdenum containing multiple component enzymes (Jimenez et al. 2008; Qiu et al. 2019; Tang et al. 2013; Yu et al. 2015). This kind of enzymes has strict substrate specificity, but they are ubiquities in microbial degradation of pyridine derivatives. Four pyridine *β*-hydroxylases have been reported (Jimenez et al. 2008; Qiu et al. 2019; Tang et al. 2013; Treiber and Schulz 2008), and the *α*-hydroxyl group in the *para*-position of the *β*-hydroxylation site is required for this kind of enzymes. Dioxygenase add two adjacent *α*-hydroxyl group and *β*-hydroxyl group to the pyridine ring, which was only reported in the microbial degradation of 2-hydroxypyridine by *Rhodococcus rhodochrous* PY11 (Vaitekūnas et al. 2015). 3-Hydroxypyridine does not contain an *α*-hydroxyl group, therefore, the first reaction cannot be pyridine *β*-hydroxylation, in which 3,5-dihydroxypyridine is produced. Hence, it is reasonable to hypothesize that the first reaction of 3-hydroxypyridine degradation is pyridine *α*-hydroxylation to form 2,3-dihydroxypyridine or 2,5-dihydroxypyridine.

**Fig. 8:**
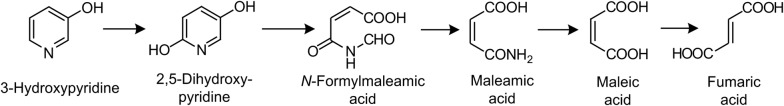
Proposed 3-hydroxypyridine degradation pathway in *Agrobacterium* sp. strain DW-1

Based on the foregoing reports, 3-hydroxypyridine could be initially metabolized to 2,3-dihydroxypyridine, 3,4-dihydroxypyridine or 2,5-dihydroxypyridine, then the dihydroxypyridine products were transformed to 2,3,6-trihydroxypyridine, which was further cleaved (Fetzner 1998; Kaiser and Bollag 1991; Kaiser et al. 1996). We detected the 2,5-dihydroxypyridine by spectrum scanning analysis and HPLC analysis; however, no 2,3-dihydroxypyridine was observed. Besides, 2,3-dihydroxypyridine could not be transformed by resting cells of strain DW-1. The results indicated that 3-hydroxypyridine was initially transformed into 2,5-dihydroxypyridine in strain DW-1. Both 2,5-dihydroxypyridine and 2,3,6-trihydroxypyridine could be the ring-cleavage intermediates during microbial degradation of pyridine derivatives (Fetzner 1998; Kaiser et al. 1996; Kost and Modyanova 1978; Tang et al. 2013). 2,3,6-Trihydroxypyridine can automatically transformed into blue pigment (Yao et al. 2012). Therefore, if 2,5-dihydroxypyridine was further transformed to 2,3,6-trihydroxypyridine by strain DW-1, the reaction mixture with cell extract was supposed to turn to blue (Tang et al. 2012, 2013). However, the blue color was not observed in the enzyme assay reactions. Instead, the absorbance at 320 nm decreased with the adding of cell extract and Fe^2+^, indicating that the ring of 2,5-dihydroxypyridine was opened by an Fe^2+^ dependent dioxygenase (Tang et al. 2013; Yao et al. 2013). Therefore, 2,5-dihydroxypyridine is the ring-cleavage intermediate during 3-hydroxypyridine degradation. Combined with the previous reports, it can be concluded that 2,5-dihydroxypyridine was oxidized to form *N*-formylmaleamic acid by Fe^2+^ dependent 2,5-dihydroxypyridine 5,6-dioxygenase. The complete 3-hydroxypyridine degradation pathway was shown in Fig. 8.

This paper reported the aerobic degradation of 3-hydroxypyridine by *Agrobacterium* sp. DW-1. The study of this paper will help us to apply this strain for 3-hydroxypyridine containing wastewater treatment and 3-hydroxypyridine related compounds degradation.

## Data Availability

The dataset supporting the conclusions of this article is included within the article. All data are fully available without restriction.
